# Supplementary material for: Impact of trypsin on cell cytoplasm during detachment of cells studied by terahertz sensing
Source: Biophys J. 2024 Jun 13;123(16):2476–83. doi: 10.1016/j.bpj.2024.06.011 (PMC11365101; doi:10.1016/j.bpj.2024.06.011)
Supplement: Document S1. Supporting material, Figures S1 and S2, and Tables S1 and S2 [file mmc1.pdf]

Biophysical Journal, Volume *123*

Supplemental information

Impact of trypsin on cell cytoplasm during detachment of cells studied by terahertz sensing

Blandine Lordon, Tiffany Campion, Laure Gibot, and Guilhem Gallot

Supplementary Information

Impact of trypsin on cell cytoplasm during detachment of cells studied by terahertz sensing,

by B. Lordon, T. Campion, L. Gibot and G. Gallot

Relationship between the terahertz permittivity and the size of the in biomolecules

Figure SI1 shows the influence of the molar mass of the molecules in solution on the terahertz ATR signal. The data and the theoretical model are taken from the Supplementary Information of [1] and from [2] for the molecules listed in Table SI1. The solid line is obtained from a 3-volume model presented in [1]. It consists in assigning a terahertz permittivity to 3 different volumes: the molecule itself, the solvation shell around the molecule and the bulk water. The data show a strong dependence of the terahertz signal on the molar mass of the molecules. The contribution is negative for the smallest molecules below about 110 Da, whereas it is positive above that, for amino acids, peptides and proteins.

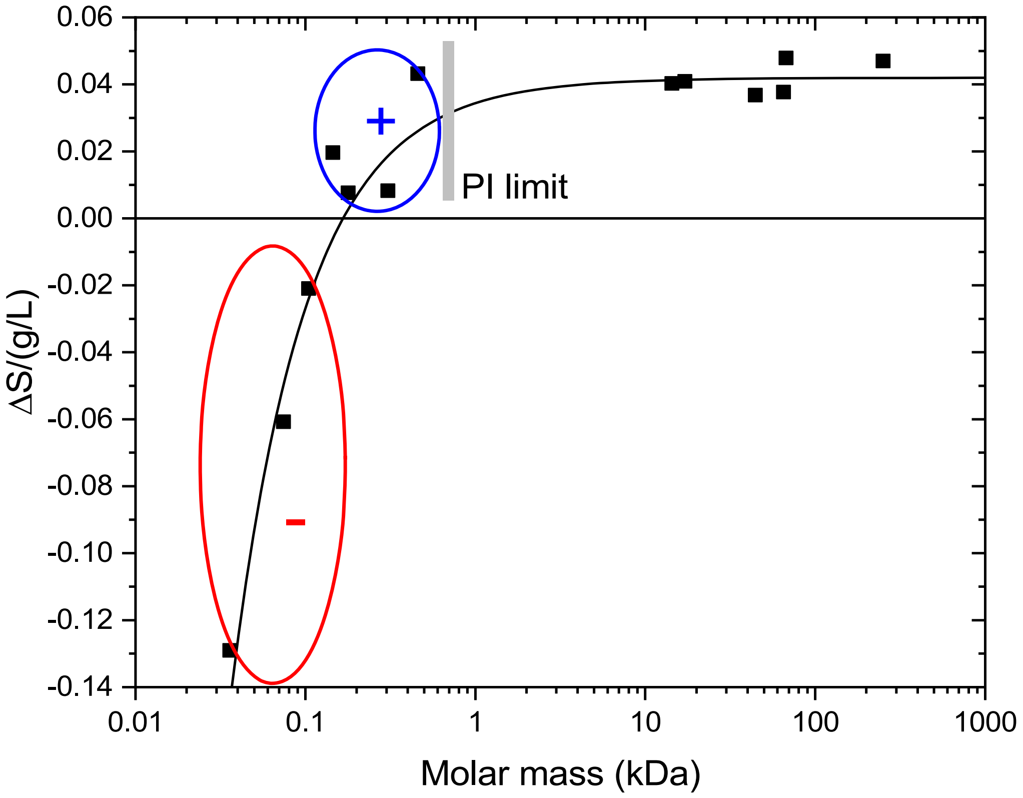

Figure SI1: Experimental and theoretical terahertz ATR reflectivity variation from biological relevant solutions found in Table SI1. ΔS/(g/L) is the molar variation of terahertz signal. The solid line is obtained from the model described in [1].

Molecule	Molar mass (kDa)
K^+/Cl^-	0.037
glycine	0.075
serine	0.105
lysine	0.146
tricine	0.179
gluthatione	0.307
LSKL-NH2 peptide	0.458
lysozyme	14.3
myoglobin	17.1
ovalbumin	44.3
hemoglobin	64.5
albumin	66
catalase	≈250

Table SI1: List of the molecules recorded in figure SI1, with their molar mass.

Contributions to the terahertz signal

The cytoplasm is experimentally found to have a positive contribution to the terahertz signal, while the PBS consists of small inorganic ions that have a negative contribution. Therefore, there are two possible explanations for a decrease in the terahertz signal, possibly at the same time. It could be due to an uptake of molecules from the PBS (see Table SI2 for the composition of the PBS) and/or an output of molecules from the cytoplasm with a molar mass between about 100 and 600 Da, as shown in Fig. SI2.

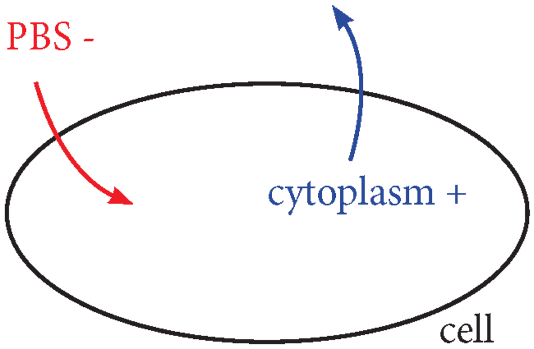

Figure SI2: Fluxes of molecules contributing to a decrease of the terahertz signal: influx from the PBS or output from the cytoplasm.

Molecules	Concentration mg/L
KH_2PO_4	144
NaCl	9000
Na_2HPO_4-$7H_2O$	795

Table SI2: Composition of PBS, pH 7.4, ThermoFisher Scientific, 10010023.

Videomicroscopy data

The monitoring by videomicroscopy of cell morphology and plasma membrane permeability (propidium iodide, red fluorescence) of MDCK1 cells incubated with trypsin (8µM) are presented in the two following movies: control and trypsin/EDTA.

Movie1-control

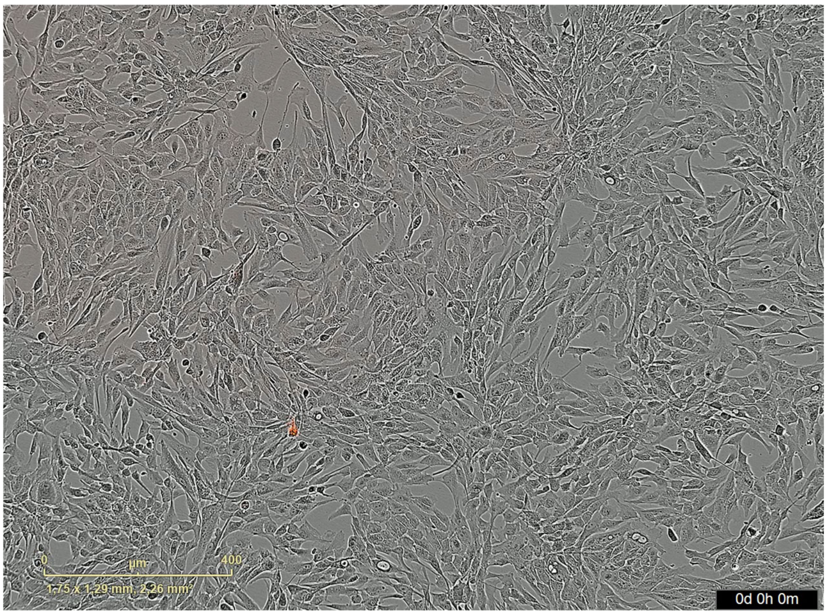

Movie1-trypsin/EDTA

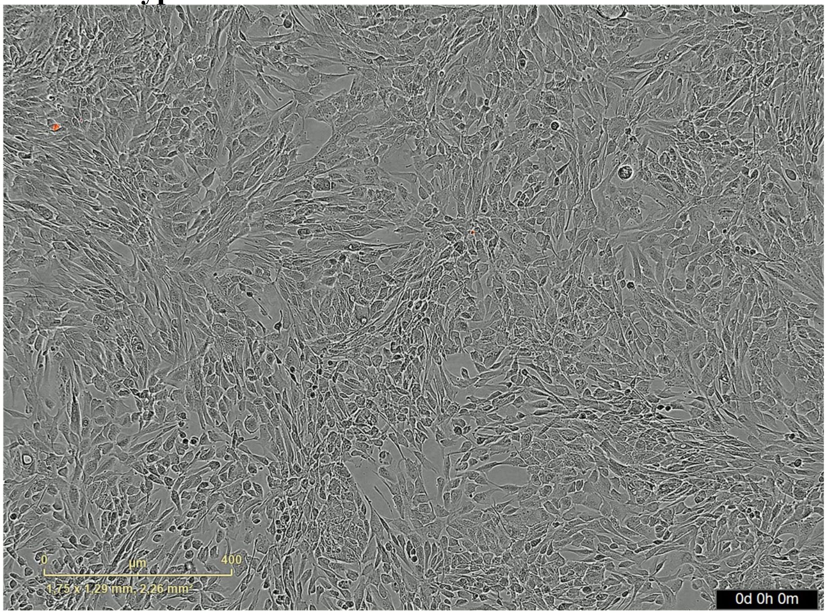

References:

[1] M. Grognot and G. Gallot, "Relative Contributions of Core Protein and Solvation Shell in the Terahertz Dielectric Properties of Protein Solutions", J. Phys. Chem. B 121, 9508 (2017).
[2] M. Grognot, "Imagerie térahertz par réflexion interne totale pour la biologie. Application à l'étude de la perméabilisation cellulaire", PhD thesis, Ecole polytechnique (2016).
