# Supplementary material for: Impact of trypsin on cell cytoplasm during detachment of cells studied by terahertz sensing
Source: Biophys J. 2024 Jun 13;123(16):2476–83. doi: 10.1016/j.bpj.2024.06.011 (PMC11365101; doi:10.1016/j.bpj.2024.06.011)
Supplement: Document S2. Article plus supporting material [file mmc3.pdf]

Biophysical Journal
Article

Biophysical Society

Impact of trypsin on cell cytoplasm during detachment of cells studied by terahertz sensing

Blandine Lordon,[1] Tiffany Campion,[2] Laure Gibot,[2] and Guilhem Gallot[1,*]
[1]LOB, Ecole Polytechnique, CNRS, INSERM, Institut Polytechnique de Paris, Palaiseau, France and [2]Laboratoire Softmat, Université de Toulouse, CNRS UMR 5623, Université Toulouse III – Paul Sabatier, Toulouse, France

ABSTRACT Trypsin is a very common enzyme used in cell culture to harvest cells by cleaving the proteins responsible for cell adhesion. However, trypsin also induces undesirable effects on cells, such as altering membrane proteins and the cytoskeleton, changing the composition of the cytoplasm and the cell volume, and even leading to cell death when used improperly. Using attenuated total reflection in the terahertz domain, confocal microscopy, and the propidium iodide test, we quantified in real time the change in cytoplasmic content induced by trypsin proteolysis on Madin-Darby canine kidney epithelial cells. We have observed a cytoplasmic modification from the very first seconds of trypsinization, following the change of cell volume due to mechanical re-equilibrium of the membrane. We found that the cytoplasmic alteration is associated with a transfer of small solutes: electrolytes and metabolites. We also found a very good nonlinear correlation between the side effects monitored by terahertz sensing and the cell height, regardless of the dependence of the cell height on trypsin concentration and exposure time.

SIGNIFICANCE Trypsin, commonly used in cell culture to detach cells by cleaving adhesion proteins, can adversely affect cells, impacting their membrane proteins, cytoskeleton, cytoplasmic composition, volume, and even viability. Research using attenuated total reflection in the terahertz domain alongside confocal microscopy has quantified these effects in real time. Results demonstrate that trypsin initiates cytoplasmic modifications almost immediately, which correlates with cell volume adjustments and membrane mechanical re-equilibrium. Furthermore, a significant nonlinear correlation was established between these side effects and cell height, independent of trypsin concentration and exposure duration. This study highlights the complex and rapid cellular responses to trypsinization, suggesting a need for careful application to preserve cell integrity in research and therapeutic contexts.

INTRODUCTION

Trypsin, a pancreatic serine protease, is used daily in biology for subculturing cells during passage or preparing cells for flow cytometry (1). Trypsin cleaves peptide bonds of proteins responsible for cell adhesion (cell-cell and cell-substrate adhesion), leaving the cells in suspension. Although highly effective for this type of process, trypsinization has numerous side effects on cells. The main reason is that trypsin undifferentiatedly hydrolyzes proteins with an arginine or lysine residue that is not followed by proline, and such termination is common to many membrane proteins. Thus, trypsin has been shown to induce many changes at the level of the cell membrane and cytoplasm. If the incubation time is too long, trypsinization may result in the loss of cell membrane proteins due to excessive enzymatic degradation, which can interfere with surface marker analysis and reduce cell viability, especially that of stem cells (2). Several groups have also shown that trypsin induces the formation of microvilli (fine, cylindrical cell extensions) on the surface of epithelial cells (3,4); this is thought to be the cell's way of dealing with excess cell membrane as the cell becomes rounded. Detachment of the cells from the substrate also induces a change in cell morphology: the cells detach from each other and from the substrate, their volume increases, and their spreading area decreases (5). Trypsin alters the cytoskeleton structure and integrity (6) that may ultimately lead to the death of the cell (5,7). The detachment of cells from the substrate also disrupts the organization of actin filaments and cortical microtubules, leading to a change in cell stiffness and osmotic balance (8).

While trypsin is widely used in cell culture, its impact on the molecular alteration of the cytoplasm has received less attention. Molecular uptake during trypsinization was

Submitted October 20, 2023, and accepted for publication June 10, 2024.
*Correspondence: guilhem.gallot@polytechnique.edu
Editor: John Conboy.
https://doi.org/10.1016/j.bpj.2024.06.011
© 2024 Biophysical Society.
This is an open access article under the CC BY license (http://creativecommons.org/licenses/by/4.0/).

observed by Lemons et al. in the late 1980s in fibroblasts using ^{125}I-BSA or [^{14}C]leucine (9). Papers have also documented trypsin-induced leakage of metabolites across the plasma membrane during treatment (10), possibly associated with cytoskeletal disorganization (11). Thus, trypsin may trigger the transfer of molecules through the membrane by several means, ion channels and pumps, exo- and endocytosis, and vesicles, resulting in a net change in the molecular composition of the cytoplasm (12–14). Understanding the dynamics of cytoplasmic modification during trypsinization is therefore interesting to minimize cell damage during the process. The techniques previously used have been mostly destructive: mass spectroscopy (15), gas chromatography and mass spectroscopy (10), radiolabeled elements and centrifugation (9), or removal of extracellular medium (16).

Many techniques are available to investigate the dynamics of the cytoplasm content in living cells: mass spectroscopy (16), gas chromatography (10), exclusion tests (17), fluorescence and Raman spectroscopy (18,19), protein assays (20), scanning electrochemical microscopy (21), bioluminescence (22), inductively coupled plasma-optical emission spectroscopy, and ion chromatography (23). The terahertz domain offers many advantages, in particular allowing quantitative and nondestructive investigation of living cells (19,24,25). Unlike the visible domain, it is directly sensitive to the content of the cytoplasm, in particular ions, metabolites, and proteins. Absorption is much less important than in the infrared, and it offers better sensitivity than the hyperfrequency region (26). Recent work has demonstrated the ability to spectroscopically study complex systems such as cells and even tissues or small organs (24,26–30). In particular, we have shown that terahertz attenuated total reflection (THz-ATR) can follow the cytoplasmic dynamics of live monolayer epithelial Madin-Darby canine kidney (MDCK1) cells in real time, without any markers, sample preparation, or destruction and with a sensitivity at least 10 times better than standard fluorescence microscopy using a propidium iodide (PI) intercalating fluorescent probe (25). Terahertz measurements are very complementary to classic measurements such as the invasive ionic, ATP, or lactate dehydrogenase (LDH) measurements used in this latest study. Therefore, we present here the terahertz response of cells during trypsinization. Using additional confocal microscopy experimental data, PI penetration monitoring by video microscopy to address plasma membrane integrity during trypsinization, and a model of the ATR/cell sensor, we show the possibility of disentangling the geometrical cell modification linked to cell detachment and cytoplasmic contributions to the measured terahertz ATR signal. The molecular exchange across the plasma membrane was then followed in real time, and dynamic parameters were studied for various trypsin concentrations. The correlation between cytoplasmic alterations and cell detachment was investigated to answer the important question of whether it is safer to use a low concentration of trypsin for a long time or reduce the exposure time to a high concentration of trypsin, in order to minimize the side effects of mandatory trypsinization for cell biology experiments.

MATERIALS AND METHODS

Cell growth

MDCK1 cells (Sigma-Aldrich, St. Louis, MO, USA, 00062106 MDCK-I) were used. MDCK1 cells are adherent cells with an epithelial morphology that grow as a monolayer. When the cells form a confluent monolayer, the thickness is approximately 7 $\pm$ 2 μm (31). The culture medium consists of Dulbecco's modified Eagle's medium (Thermo Fisher Scientific, Waltham, MA, USA, 10566016) supplemented with 10% fetal bovine serum (Thermo Fisher Scientific, 10500064) and 1% penicillin-streptomycin (Thermo Fisher Scientific, 15140122). To pass the MDCK1 cells, we expose them to a concentration of 10 μM (i.e., 250 mg/L) of trypsin/ethylenediaminetetraacetate acid (EDTA; Thermo Fisher Scientific, R001100) for 7 min. The cells were then seeded onto either glass coverslips (for confocal measurements) or silicon plates (for terahertz measurements). The cell seeding density was 31,250 per cm^2, counted with a Malassez hemocytometer. Cells were grown to confluence for 48 h and washed twice with phosphate-buffered saline (PBS) buffer (Thermo Fisher Scientific, 10010023). Before terahertz experiments, the cells are scraped from one half of the silicon plate to provide a reference signal during the terahertz measurements.

Trypsin/EDTA experiments

Trypsin/EDTA is a combination of trypsin, a protease that cleaves the peptide bonds of proteins, and EDTA, a calcium and magnesium chelator. EDTA is added to trypsin to improve its efficiency in weakening cell-cell adhesion and increase trypsin's access to peptide bonds targeted for hydrolysis. In the different experiments, we dilute trypsin/EDTA with PBS to reach the required concentrations of 0.5–8 μM. As a digestive enzyme, trypsin exhibits optimal activity at 37°C (32).

PI penetration

Plasma membrane defects were visualized using PI (Merck #P4170, Darmstadt, Germany) thanks to video microscopy. PI is a nonpermeant fluorescent DNA intercalant, meaning penetration occurs only inside cells presenting loss of plasma membrane integrity. Briefly, 20,000 MDCK1 cells grown for 48 h in 96-well plates until reaching 95% confluency were washed twice with PBS without Ca^{2+} and Mg^{2+} and then incubated with 100 μL of trypsin/EDTA (8 μM) containing 1 μM PI. Plates were then immediately placed within an IncuCyte S3 (Sartorius, Göttingen, Germany). Pictures in phase and red fluorescence were acquired every 5 min over 2 h, with quantification performed using software linked to the video microscope. Video S1 shows the cell morphological aspect and penetration of PI into cells during incubation with trypsin.

THz-ATR measurements

A vertically polarized 3 mW, 2.5 THz continuous-wave beam (Lytid, Paris, France, TeraCascade 1000), collimated to 4 mm full width at half maximum, is passed through the prism made of high-resistivity silicon (HR-Si, n = 3.42) in an ATR configuration (see Fig. 1). An evanescent wave extends at the interface between the top of the prism and the sample. Provided that the thickness of the cell layer matches the penetration depth of the evanescent wave, the reflected terahertz wave is correlated with the terahertz relative permittivity of the cell layer in contact with the top of

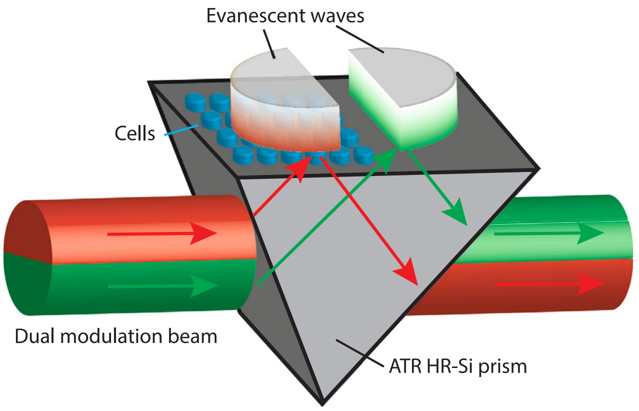

FIGURE 1 THz-ATR device. Two modulated beams arrive on the HR-Si prism in ATR configuration. Both beams induce an evanescent field above the prism (*red and green gradient areas*). A confluent layer of epithelial cells is placed in one of the evanescent fields. The other evanescent field is used as a reference. The reflected beam then takes into account the changes in the evanescent field induced by the sample, normalized to that of the reference. To see this figure in color, go online.

the prism. The origin of the terahertz contrast was found to be related to the modification of the dielectric constant of liquid water in the presence of solutes such as ions, peptides, or proteins (33). In our experiments, the thickness of the cell layer is 7 $\pm$ 2 μm, which agrees well with a penetration depth of 8 μm at 2.5 THz. The THz-ATR sensor then allows real-time and continuous measurement of the change in the cell cytoplasm concentration with a time resolution of a few seconds. To achieve a high stability of the measurement, a dual frequency optical chopper splits the main beam into two halves, which are chopped simultaneously at two different frequencies (24). The upper and lower halves of the beam are modulated, relative to a master frequency set at 65 Hz, at frequencies of $\times$ 6 (390 Hz) and $\times$ 5 (325 Hz), respectively. Both halves are then subjected to ATR on the HR-Si prism, in two separate positions, where the cell sample and reference liquid are placed. After leaving the prism, both parts of the parallel beam are focused on a pyroelectric detector (Lytid, TeraPyro). The pyroelectric signal, resulting from the superposition of the two modulations, is demodulated by two independent lock-in amplifiers driven by each of the two frequencies provided by the chopper controller (SRS, Sunnyvale, CA, USA, SR540). One obtains the signals S_5 ($\times$ 5) and S_6 ($\times$ 6) for both modulations. The whole setup is placed in a sealed box with controlled humidity (below 1% relative humidity) and temperature (21°C $\pm$ 0.5°C). The ATR prism is also precisely thermalized at 37°C $\pm$ 0.01°C using thermoelectric Peltier coolers and a temperature controller (Thorlabs, Newton, NJ, USA, TED200C). To cancel out the residual fluctuations, the THz signal S_{THz} used for the measurements is calculated as $S_{THz} = S_5/S_6$. The THz-ATR sensor is therefore characterized by an excellent signal/noise ratio and long-term stability ($< 10^{-3}$). Since the ATR prism cannot be easily removed, the MDCK1 cells were grown on a separate 3-mm-thick HR-Si plate, which is placed on top of the ATR prism. The area probed by the evanescent field measures around 20 mm^2. A small drop of α-pinene (Sigma-Aldrich, P45702) is used as an index matching layer to ensure optical continuity between the prism and the cell plate.

Confocal measurements

We used a confocal microscope (Leica, Wetzlar, Germany, TCS SP8 X) to obtain the volume of the monolayer of confluent MDCK1 cells. Cell labeling was performed with fluorescent CellTracker Orange CMTMR (Thermo Fisher Scientific, C2927). It freely passes through the cell plasma membrane into the cytoplasm, where it is converted into plasma membrane impermeant molecules. It is stable and nontoxic under working conditions. The excitation peak is at 552 nm, and the emission peak is at 580 nm. The final dye concentration was 0.5 μM, diluted in a solution of PBS. For staining, the culture medium was removed, the CellTracker Orange solution was added, and the cells were incubated for 35 min at 37°C with a 5% CO_2 controlled atmosphere. Afterward, the slips were then washed twice with PBS, placed on a plate holder, and covered with the PBS solution. During confocal imaging, the temperature was set at 37°C. The field of view was 291.2 $\times$ 291.2 μm (0.085 mm^2); stacks were acquired with a height step of 0.5 μm over a range of 20 μm. First, we acquired a reference volume of the cell layer. Then, trypsin/EDTA was added at the required concentration, and we recorded an XYZ volume of the cell layer every minute for 15 min.

RESULTS AND DISCUSSION

We studied the phenomenon of trypsin proteolysis using the terahertz sensor with the aim of quantifying trypsin-induced cytoplasmic content dynamics. We hypothesized that the terahertz signal depends on two different contributions: a contribution resulting from induced morphological changes because of progressive cell detachment and a contribution resulting from cytoplasmic modification because of hypothetical plasma membrane defects caused by enzymatic (over)activity (duration of incubation, concentration, etc.). For measurements with the THz-ATR sensor, a silicon plate with confluent MDCK1 cells was placed on the HR-Si prism and covered with 1 mL PBS. The terahertz signal R_{THz} was recorded for 15 min to check the stability and let the temperature of the medium stabilize at 37°C and for normalization purposes ($t < 0$, *blue zone*, Fig. 2 *A*). At $t = 0$, a concentrated solution of trypsin/EDTA in PBS was added to reach the required trypsin concentration. The solution was pumped in and out several times to homogenize the solution around the cells. We then recorded the THz-ATR signal for an additional 30 min (*green zone*, Fig. 2 *A*). Finally, the cells were scraped off the silicon plate to provide a reference for normalization (*yellow zone*, Fig. 2 *A*). We chose trypsin concentrations between 0.5 and 8 μM, corresponding to the lower range of concentrations classically used in cell culture. However, the dynamics directly obtained by the THz-ATR sensor potentially takes into account both the cytoplasm and the morphological contributions since the terahertz sensing by the evanescent wave is localized close to the ATR prism surface.

Therefore, to determine the morphological contribution induced by trypsin proteolysis, we used confocal microscopy on confluent MDCK1 cells. Cells were grown and stained with CellTracker Orange. They were then placed on a plate holder and covered with 1 mL PBS and exposed to a controlled atmosphere at 37°C. A reference volume was acquired with the confocal microscope using a 0.5 μm z-step. A concentrated solution of trypsin/EDTA in PBS was then added to the medium surrounding the cells to achieve the required trypsin concentration from 1 to 8 μM Fig. 2 *B*1 shows the reference stack cross section and

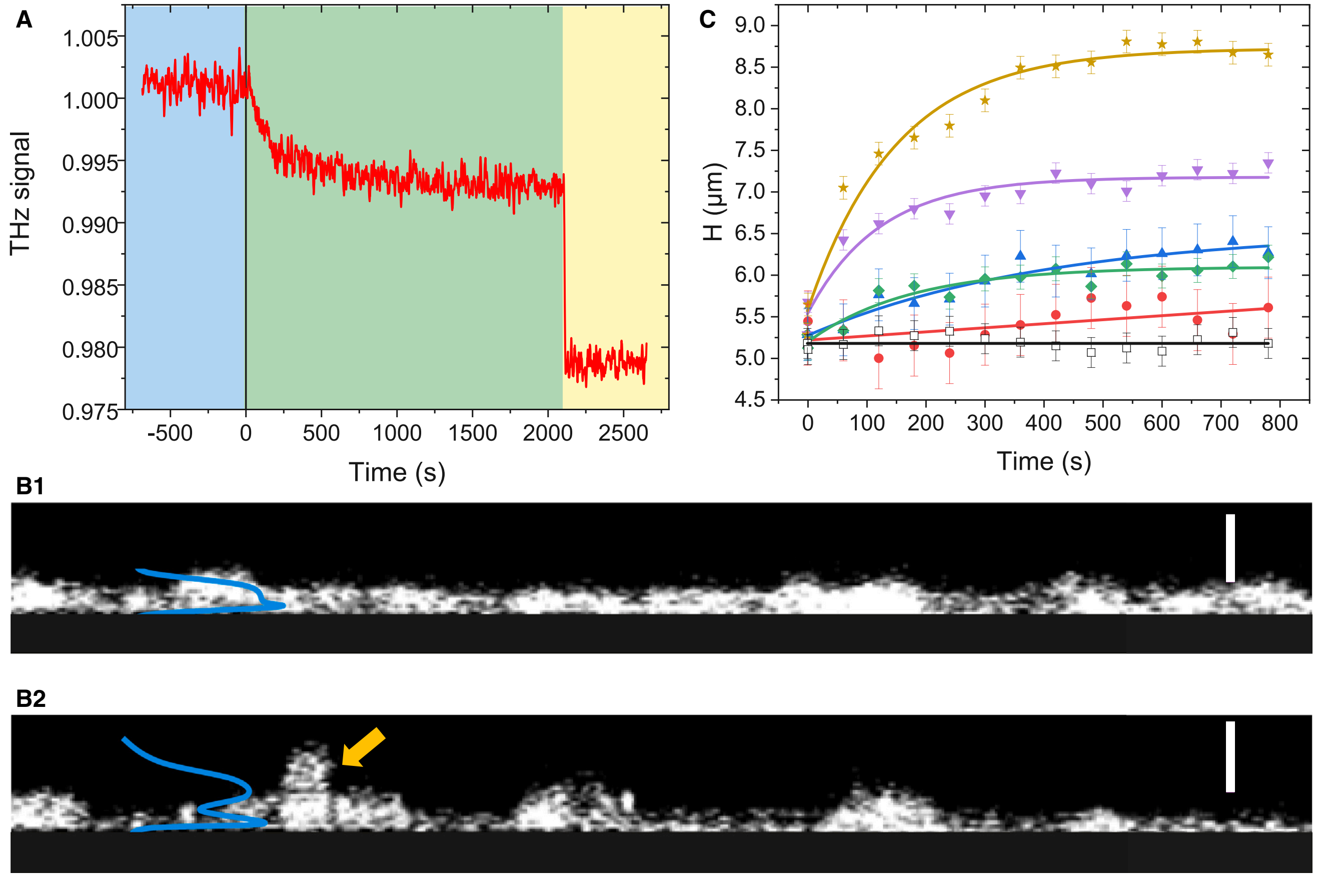

FIGURE 2 Experimental data. (*A*) THz-ATR acquisition during trypsinization of MDCK1 cells with a trypsin/EDTA concentration of 5 μM. The signal is divided into 3 zones. The left blue zone is a stabilization phase in PBS; the middle green zone is the dynamic of the signal during trypsin proteolysis, with trypsin/EDTA being added at $t = 0$; and the right yellow zone is the signal after scraping the cells off the silicon plate. (*B*) Confocal microscopy on MDCK1 cells, transverse view. (*B*1) Layer of confluent MDCK1 cells stained with CellTracker Orange. (*B*2) Layer of the same cells but trypsinized for 15 min with a concentration of 5 μM. The blue curve shows the dye intensity distribution. The yellow arrow indicates a cell that detached from the substrate. The scale bar is 20 μm. (*C*) Evolution of the thickness of the MDCK1 cell layer as a function of time during trypsinization for different concentrations evaluated by confocal microscopy. The dots are the measured width of the intensity distribution of the CellTracker Orange dye staining the cytosol. The solid lines correspond to an exponential growth fit. The black curve is the control; red = 1 μM; blue = 2 μM; green = 3.5 μM; purple = 5 μM; orange = 8 μM. To see this figure in color, go online.

Fig. 2 *B*2 the trypsinized cells for a concentration of 5 μM for 15 min. The intensity distribution of the fluorophore along the z-direction is then calculated by summation. A double Gaussian fit is applied to the intensity distribution. The first Gaussian is narrow and located close to the microscope slide, corresponding to residual dye molecules at the slide interface. This peak is found to be stationary along the trypsinization. In contrast, the second peak is much larger and is related to the cells that detach from the slide. It gives the averaged cell height $H(t)$ along z. This height is calculated at each time interval ($\Delta t = 1$ min) for 15 min (see Fig. 2 *C*). It corresponds to the morphological contribution of trypsin proteolysis on the MDCK1 cells. The smallest change corresponds to a trypsin concentration of 1 μM. Starting from 2 μM, the cell height increases rapidly during the first minutes after trypsin addition and then reaches a plateau after about 10 min. Exponential growth functions fit the data well, as shown by the solid lines in Fig. 2 *C*. These changes in cell morphology, i.e., round shaping and detachment, were also seen by video microscopy (Video S1).

To analyze the terahertz data, and to disentangle the geometrical and cytoplasmic contributions, we model the experimental setup (see Fig. 3) as a multilayer system: the HR-Si prism, the cell monolayer, and the extracellular medium considered as infinite above the cells. Each layer is characterized by its optical relative permittivity at 2.5 THz. The reflection coefficient R from the ATR prism is calculated for p-polarization, corresponding to the laser polarization, from the Fresnel reflection coefficient of a multilayer optical system (34). It depends on the permittivity and the thickness and confluency of the cell layer and is then potentially modified by trypsin/EDTA proteolysis. Previous studies showed that the permittivities of the solutes in the terahertz range mainly depend on their molar mass M, so the cytoplasm permittivity is the average of the

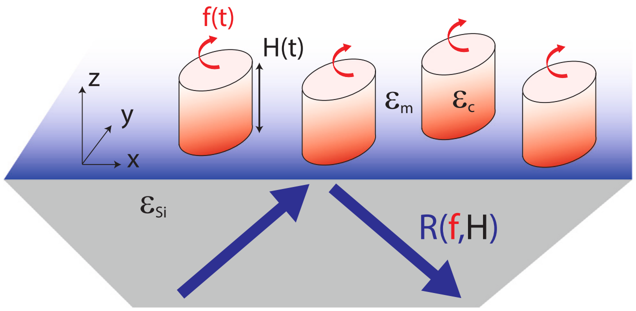

FIGURE 3 Multilayer model to compute the reflection coefficient as a function of the height of the cell layer $H(t)$ and the fraction of cytoplasm content variation $f(t)$. The blue arrows show the incident and reflected terahertz beams. The red arrows show the change of cytoplasmic content. The blue gradient represents the evanescent field. To see this figure in color, go online.

permittivity of each solute, weighted by its terahertz response (33,35).

The parameters of the model are introduced in Table 1. We define the cytoplasm and medium complex permittivities by ϵ_c and ϵ_m, respectively. The difference $\Delta\epsilon = \epsilon_c - \epsilon_m$ is obtained by

$$\Delta\epsilon(t) = \frac{1}{V_c(t)} \int D(t,M)\, \alpha(M)\, \mathrm{d}M = \frac{\alpha_T(t)}{V_c(t)}, \quad \text{(Equation 1)}$$

where V_c is the probed volume of cytoplasm, α is the solute polarizability, D is the molar distribution of the solutes in the cytoplasm, and α_T is the total polarizability. Then, we define the fraction of cytoplasm content variation f from the physiological conditions at time $t = 0$ by $\alpha_T(t) = f(t)\, \alpha_T(0)$.

We now introduce ϵ_{eff}, the complex permittivity of the area probed by the evanescent wave. Before the addition of trypsin/EDTA at time $t = 0$, ϵ_{eff} is a function of the total surface probed S_T and the complex permittivities of the medium and the cytoplasm: ϵ_m and ϵ_c, respectively. Using the Gladstone-Dale equation (36), the effective permittivity $\epsilon_{\text{eff}} = \frac{1}{S_T}[\epsilon_c S_c + \epsilon_m S_m]$, where S_c and S_m are the respective probed surfaces of the cytoplasm and medium, satisfying the relationship $S_m + S_c = S_T$. Thus, $\epsilon_{\text{eff}} = \frac{1}{S_T}[\epsilon_c S_c + \epsilon_m(S_T - S_c)]$.

Using Eq. 1 and the relationship $V_c = H \cdot S_c$, where H is the cell layer height, one obtains

TABLE 1 List of Parameters

Parameter	Symbol
Cell height	H
Cytoplasm, medium, and total probed surfaces	S_c, S_m, S_T
Probed volume of cytoplasm	$V_c = H \cdot S_c$
Cytoplasm, medium, silicon, and effective permittivity	$\epsilon_c, \epsilon_m, \epsilon_{\text{Si}}, \epsilon_{\text{eff}}$
Fraction of cytoplasm content variation	f

$$\begin{aligned} \epsilon_{\text{eff}} &= \frac{1}{S_T}\left[S_c\left(\epsilon_m + f(t)\frac{\alpha_T(0)}{V_c(t)}\right) + \epsilon_m(S_T - S_c)\right] \\ &= \epsilon_m[1 + \delta(t)] \text{ with } \delta(t) = f(t)\frac{\alpha_T(0)}{\epsilon_m H(t) S_T}. \end{aligned} \quad \text{(Equation 2)}$$

This result may seem surprising since ϵ_{eff} does not depend explicitly on cell volume but only on two parameters: the change in cytoplasm $f(t)$ and the height of the cell layer $H(t)$. This is due to the particularity of the terahertz device, which is based on the evanescent field along the direction of the z-axis at the surface of the prism, which breaks the symmetry of the volume (see Fig. 3). This means that the terahertz signal remains constant when the cells are spread out as long as the solute molecules remain at the same distance from the prism surface, i.e., along the (xy) plane.

Going back to the model and using the Fresnel equation for a multilayer system (34), we compute the reflection coefficient from the ATR prism R_{THz} as the product between R_0 and a function of H, ϵ_{Si}, ϵ_{eff}, and ϵ_m. The proportionality coefficient R_0 takes into account the unknown constant transmission coefficient of the whole system apart from the ATR prism.

The permittivity of silicon is known, $\epsilon_{Si} = 11.7$ (37), and the one of the medium is very close to the one of water and also well known (38). The normalization procedure is in two steps. First, R_0 is obtained from empty cell data, where $f = 0$ (see *zone 3*, *yellow*, in Fig. 2 *A*). Second, R_{THz} is compared to the experimental data without trypsin/EDTA (*zone 1*, *blue*, in Fig. 2 *A* at $t < 0$) where $f = 1$. We compute δ_0 in ϵ_{eff} so that $R(\delta_0) = R_{\text{THz}}(t < 0)$, and then the effective permittivity is written

$$\epsilon_{\text{eff}} = \epsilon_m\left[1 + \delta_0 f(t)\frac{H_0}{H(t)}\right], \quad \text{(Equation 3)}$$

where H_0 is the cell height at rest without trypsin/EDTA. Therefore, the only remaining unknown parameters are $f(t)$ and $H(t)$. Finally, we can discuss the influence of the molar mass of the molecules involved in the terahertz signal, taking into account the variation of the solute polarizability α in the effective permittivity (Eq. 1) and, subsequently, in R_{THz}. As described in more details in the supporting material (Fig. S1), small solutes such as the one found in PBS have a negative contribution to the measured terahertz signal. On the contrary, it is positive for bigger molecules such as proteins.

We now introduce $R_p(x, H)$, the theoretical reflected signal calculated using the model described in the materials and methods section; its inputs are the height of the MDCK1 layer H and the fraction of cytoplasm content f (see Fig. 3). Knowing $H(t)$ from the confocal measurements, we can calculate $f(t)$ such that $R_{THz} = R(f,H)$ for each trypsin concentration. Raw terahertz measurements were used for R_{THz}, while exponential fits were used for $H(t)$ to minimize

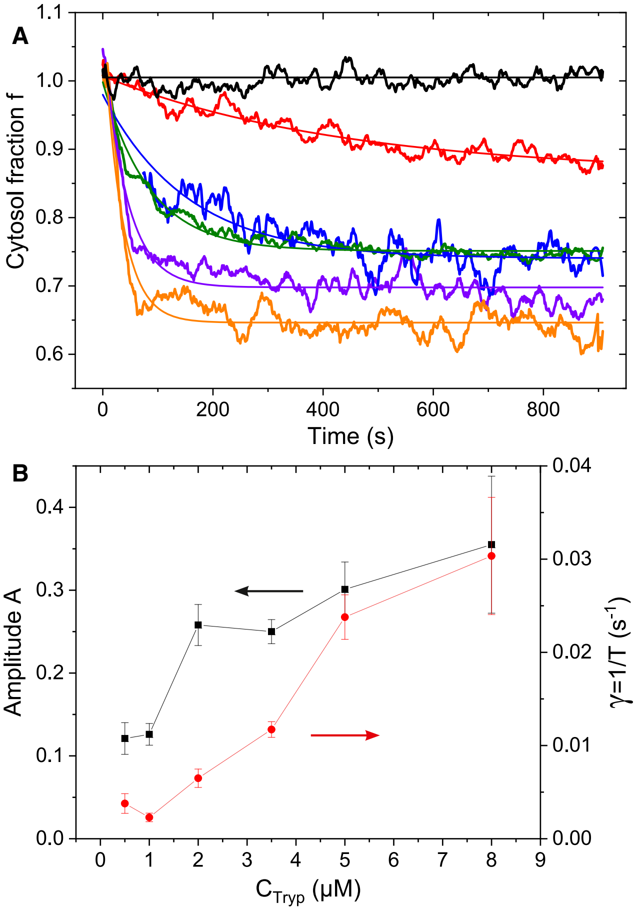

FIGURE 4 Dynamics of cell cytoplasm content. (*A*) Fraction of cytoplasm content $f(t)$ for different concentrations of trypsin/EDTA. The black curve is the control (3); red = 1 μM (6); green = 3.5 μM (3); purple = 5 μM (5); orange = 8 μM (3). () is the number of samples. (*B*) Parameters of the exponential decay fit of $f(t)$ for the different trypsin/EDTA concentrations $f_0 + A\,e^{-t/T}$. The black square curve represents the amplitude A of the decay; the red curve is the decay rate $\gamma = 1/T$. To see this figure in color, go online.

fluctuations. The evolution of $f(t)$ is shown in Fig. 4 *A*. We observe a decay of f in the first 200 s, followed by a plateau. The contribution of the geometrical contribution to the total terahertz signal is about 25% for total cell detachment. The dynamics are typical exponential behavior. A monoexponential decay fit $f_0 + A\,e^{-t/T}$ was performed to obtain the amplitude of the decay A and the characteristic decay time T. Fig. 4 *B* shows the evolution of A and the decay rate $\gamma = 1/T$ versus trypsin concentration. We observe an important increase of both A and γ with trypsin concentration. The modification of cytoplasm is weak for the lower concentration at 0.5 μM, corresponding to about 10% of the variation in cytoplasm content. It reaches 30% for a trypsin concentration of 8 μM. The magnitude of the changes in the characteristic decay time is even larger, by a factor 10. It ranges from 33 $\pm$ 5 s at 8 μM to 320 $\pm$ 50 s at 1 μM concentrations.

Since f is only due to a change in the number of molecules in the cytoplasm, molecular transfer may occur in and out of the cells. We monitored the penetration of PI by confocal video microscopy (see Fig. 5), which showed negligible PI entry, at least 100 times lower than the control with saponin, the reference for creating pores in the plasma membrane (39). This test excludes the permeabilization of molecules bigger than PI (690 Da). However, Dettmer et al. (10) observed significant metabolite leakage in the SW480 adenocarcinoma cell line after trypsin/EDTA treatment using gas chromatography-mass spectrometry. All these metabolites have a mass of between 90 and 340 Da. They also observed the release of the amino acids alanine and ornithine with a characteristic time of about 10 min, which is consistent with that observed for f (Fig. 4 *B*). Furthermore, the change in cell volume observed during trypsinization is associated with cell membrane expansion and the flattening of microvilli (finger-like projections with actin cores) or membrane folds and with a change in membrane stiffness and actin microfilament structure (40). This unfolding is also balanced by endo- and exocytosis (41,42). Venkova et al. (43) demonstrated the existence of a mechano-osmotic coupling that defines a membrane tension homeostasis operating in cells, causing volume fluctuations associated with rapid changes in cell shape, with potential consequences for cell physiology. Volume variations of up to 20% have been observed, implying a homeostatic response of the cell and, therefore, a transfer of electrolytes across the cell membrane. These observations can explain the observed decrease in the terahertz signal. Firstly, the entry into the cytoplasm of ions from the PBS solution surrounding the cells leads to a decrease in the signal since the contribution of these small inorganic ions is negative, as previously discussed (see also the supporting material; Figs. S1 and S2). Secondly, the exit of amino acids and small peptides from

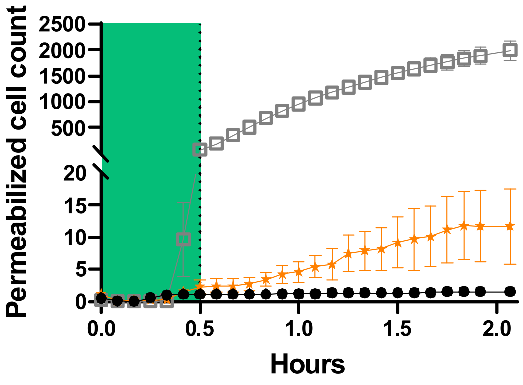

FIGURE 5 Monitoring the penetration of propidium iodide by video microscopy after incubation of MDCK1 cells with 8 μM trypsin (*orange star* ★). The green zone represents the dynamics of the signal during the first 30 min of exposure to trypsin proteolysis, as monitored by terahertz radiation in Fig. 2. Saponin detergent (*gray square* □) is used as a positive control for membrane permeabilization. The negative control (no trypsin) is shown as black dots ●. For each, n = 5. To see this figure in color, go online.

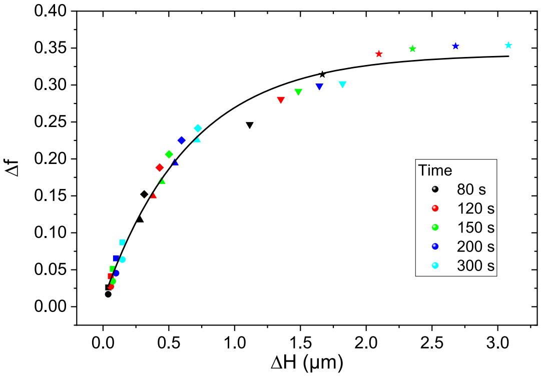

FIGURE 6 Correlation between the fraction of cytoplasm and the cell height. Cytoplasm content variation Δf versus cell height variation ΔH for five identical time delays from 80 to 300 s and for six trypsin/EDTA concentrations. The dot colors refer to the time delays as indicated in the legend. The dot shapes refer to concentrations at 0.5 (*circle* ●), 1 (*square* ■), 2 (*up triangle* ▲), 3.5 (*diamond* ◆), 5 (*down triangle* ▼), and 8 μM (*star* ★). The solid line is an exponential growth function fit $\Delta f = 0.332(1 - e^{-\Delta H/0.655})$. To see this figure in color, go online.

the cytoplasm also leads to a decrease in signal since their contribution to the signal is positive. The molar mass of such molecules, as determined by Dettmer et al. (10), is less than 240 Da and is therefore consistent with the PI exclusion tests (Fig. 5).

We then further investigate the following question: is it safer to use a high concentration of trypsin for a short time or increase the exposure time to a low concentration of trypsin, in order to minimize the cytoplasmic side effects of trypsinization? In other words, what is the intrinsic role of trypsin concentration on cytoplasmic alteration side effects? We proceed as follows. At each investigated trypsin/EDTA concentration and from the recorded dynamics of $x(t)$ and $H(t)$, we can calculate for a given time delay t_1 the corresponding values of cell height variation $\Delta H = H(t_1) - H_0$ and the cytoplasm content variation $\Delta x = 1 - x(t_1)$. The results are shown in Fig. 6 for five time delays from 80 to 300 s and for six concentrations from 0.5 to 8 μM and show a remarkable nonlinear correlation between Δx and ΔH ($R^2 \approx 0.98$) with the fitting equation $\Delta f = 0.332(1 - e^{-\Delta H/0.655})$. The points define a regular curve independent of the concentration. This means that when a given cell detachment is obtained during trypsinization, the cytoplasm modification is always the same regardless of the trypsin/EDTA concentration used, at least in the concentration range used in this study. Therefore, the same cytoplasmic side effect is expected whether a low concentration of trypsin is used for a long time or a high concentration is used for a short time. It is interesting to note that, in the range of trypsin concentrations studied here, the impact on the cell depends essentially on the total number of trypsin molecules encountered by the cell, independent of the temporal arrival of these molecules. This implies that the effect of trypsin/EDTA seems to be very tolerant in the sense that a reasonably large range of trypsin concentrations can be used to achieve the same results on the cells. This is in agreement with the cell culture procedures.

CONCLUSION

In this article, the modification of the cytoplasmic content as a side effect of cell detachment during trypsinization was studied in real time by THz, confocal microscopy, and video microscopy measurements. Using the geometric data obtained from confocal microscopy and a model of the cell/sensor interaction, THz-ATR sensing proved to be a very interesting technique to obtain real-time quantitative data on cytoplasmic dynamics due to the action of trypsin/EDTA on the membrane proteins. A significant modification of the cytoplasmic content was observed during the first minutes after the addition of trypsin/EDTA, whose amplitude and dynamic rate increase sharply with the trypsin concentration. This modification can be explained by both the entry of inorganic ions from the extracellular PBS solution and the leakage of amino acids and small peptides from the cytoplasm. Interestingly, we found a very good nonlinear correlation between the cytoplasm alteration and the cell height, regardless of the dependence of the cell height on trypsin concentration and exposure time. This may explain why the cell culture procedures found in the literature are tolerant to the concentrations to be used.

SUPPORTING MATERIAL

Supporting material can be found online at https://doi.org/10.1016/j.bpj.2024.06.011.

AUTHOR CONTRIBUTIONS

B.L. carried out the terahertz experiments. T.C. performed the video microscopy measurements. B.L. and G.G. carried out the modeling and calculations. B.L., L.G., and G.G. wrote the article. G.G. designed the research.

ACKNOWLEDGMENTS

This research was funded by the French Agence Nationale de la recherche (ANR-11-EQPX-0029 and ANR-21-CE42-0018).

DECLARATION OF INTERESTS

The authors declare no competing interests.

REFERENCES

1. Campbell, N. 2016. Biology, 11th edition. Pearson.
2. Fischer, B., A. Meier, …, L. Gentile. 2018. A Complete Workflow for the Differentiation and the Dissociation of hiPSC-derived Cardiospheres. *Stem Cell Res.* 32:65–72.

3. Harrison, C. J., and T. D. Allen. 1979. Cell Surface Morphology After Trypsinisation Depends on Initial Cell Shape. *Differentiation.* 15:61–66.
4. Furcht, L. T., and G. Wendelschafer-Crabb. 1978. Trypsin-Induced Coordinate Alterations in Cell Shape, Cytoskeleton, and Intrinsic Membrane Structure of Contact-Inhibited Cells. *Exp. Cell Res.* 114:1–14.
5. Yan, J., C. Xie, …, L. Li. 2021. Effect of Trypsin Concentration on Living SMCC-7721 Cells Studied by Atomic Force Microscopy. *J. Microsc.* 284:203–213.
6. Raizada, M. K., G. Tan, and R. E. Fellows. 1981. Trypsin-induced alterations of insulin binding, microfilament organization and cell shape in fibroblastic cultures from non-diabetic and diabetic mice. *Exp. Cell Res.* 133:261–271.
7. Nowak-Terpilowska, A., P. Sledzinski, and J. Zeyland. 2021. Impact of Cell Harvesting Methods on Detection of Cell Surface Proteins and Apoptotic Markers. *Braz. J. Med. Biol. Res.* 54:e10197.
8. Nehls, S., H. Nöding, …, A. Janshoff. 2019. Stiffness of MDCK II Cells Depends on Confluency and Cell Size. *Biophys. J.* 116:2204–2211.
9. Lemons, R., S. Forster, and J. Thoene. 1988. Protein Microinjection by Protease Permeabilization of Fibroblasts. *Anal. Biochem.* 172:219–227.
10. Dettmer, K., N. Nürnberger, …, P. J. Oefner. 2011. Metabolite Extraction from Adherently Growing Mammalian Cells for Metabolomics Studies: Optimization of Harvesting and Extraction Protocols. *Anal. Bioanal. Chem.* 399:1127–1139.
11. Liu, X., S. Oh, and M. W. Kirschner. 2022. The Uniformity and Stability of Cellular Mass Density in Mammalian Cell Culture. *Front. Cell Dev. Biol.* 10:1017499.
12. Cadart, C., L. Venkova, …, M. Piel. 2019. The Physics of Cell-Size Regulation across Timescales. *Nat. Phys.* 15:993–1004.
13. Hapala, I. 1997. Breaking the Barrier: Methods for Reversible Permeabilization of Cellular Membranes. *Crit. Rev. Biotechnol.* 17:105–122.
14. Stewart, M. P., A. Sharei, …, K. F. Jensen. 2016. In Vitro and Ex Vivo Strategies for Intracellular Delivery. *Nature.* 538:183–192.
15. Ziemanski, J. F., J. Chen, and K. K. Nichols. 2020. Evaluation of Cell Harvesting Techniques to Optimize Lipidomic Analysis from Human Meibomian Gland Epithelial Cells in Culture. *Int. J. Mol. Sci.* 21:3277.
16. Vogel, K. G. 1978. Effects of Hyaluronidase, Trypsin, and EDTA on Surface Composition and Topography during Detachment of Cells in Culture. *Exp. Cell Res.* 113:345–357.
17. Stoddart, J. M. 2011. Mammalian Cell Viability: Methods and Protocols. Humana Press.
18. Rols, M. P., and J. Teissié. 1990. Electropermeabilization of Mammalian Cells. Quantitative Analysis of the Phenomenon. *Biophys. J.* 58:1089–1098.
19. Azan, A., M. Grognot, …, L. M. Mir. 2020. Monitoring the Molecular Composition of Live Cells Exposed to Electric Pulses via Label-Free Optical Methods. *Sci. Rep.* 10:10471.
20. Walker, J. M. 2009. The Protein Protocols Handbook. Springer Protocols Handbooks. Humana Press.
21. Koley, D., and A. J. Bard. 2010. Triton X-100 concentration effects on membrane permeability of a single HeLa cell by scanning electrochemical microscopy (SECM). *Proc. Natl. Acad. Sci. USA.* 107:16783–16787.
22. Champiat, D., A. Roux, …, G. Nosenzo. 1994. Biochemiluminescence and Biomedical Applications. *Cell Biol. Toxicol.* 10:345–351.
23. Wouters, B., C. Bruggink, …, S. Eeltink. 2012. Capillary Ion Chromatography at High Pressure and Temperature. *Anal. Chem.* 84:7212–7217.
24. Grognot, M., and G. Gallot. 2015. Quantitative Measurement of Permeabilization of Living Cells by Terahertz Attenuated Total Reflection. *Appl. Phys. Lett.* 107:103702.
25. Zheng, X., B. Lordon, …, G. Gallot. 2023. Terahertz Spectroscopy Sheds Light on Real-Time Exchange Kinetics Occurring through Plasma Membrane during Photodynamic Therapy Treatment. *Adv. Sci.* 10
26. Masson, J. B., M. P. Sauviat, …, G. Gallot. 2006. Ionic Contrast Terahertz near Field Imaging of Axonal Water Fluxes. *Proc. Natl. Acad. Sci. USA.* 103:4808–4812.
27. Shiraga, K., Y. Ogawa, …, M. Imamura. 2014. Characterization of Dielectric Responses of Human Cancer Cells in the Terahertz Region. *J. Infrared, Millim. Terahertz Waves.* 35:493–502.
28. Yu, C., S. Fan, …, E. Pickwell-MacPherson. 2012. The Potential of Terahertz Imaging for Cancer Diagnosis: A Review of Investigations to Date. *Quant. Imag. Med. Surg.* 2:33–45.
29. Woodward, R. M., B. E. Cole, …, M. Pepper. 2002. Terahertz pulse imaging in reflection geometry of human skin cancer and skin tissue. *Phys. Med. Biol.* 47:3853–3863.
30. Masson, J.-B., M.-P. Sauviat, and G. Gallot. 2006. Ionic Contrast Terahertz Time Resolved Imaging of Frog Auricular Heart Muscle Electrical Activity. *Appl. Phys. Lett.* 89:153904.
31. Cho, M., and D. Thompson. 1987. The Madin Darby Canine Kidny (MDCK) epithelial cell monolayer as a model cellular - transport barrier. *J. Pharmaceut. Sci.* 76:S49.
32. Sipos, T., and J. R. Merkel. 1970. An effect of calcium ions on the activity, heat stability, and structure of trypsin. *Biochemistry.* 9:2766–2775.
33. Grognot, M., and G. Gallot. 2017. Relative Contributions of Core Protein and Solvation Shell in the Terahertz Dielectric Properties of Protein Solutions. *J. Phys. Chem. B.* 121:9508–9512.
34. Born, M., and E. Wolf. 1980. Chapter II - Electromagnetic Potentials and Polarization, Sixth Edition. Principles of Optics, pp. 71–108.
35. Zheng, X., T. Gevart, and G. Gallot. 2021. High Precision Dual-Modulation Differential Terahertz ATR Sensor for Liquid Measurements. *Opt. Lett.* 46:4045–4048.
36. Reis, J. C. R., I. M. S. Lampreia, …, G. Douhéret. 2010. Refractive Index of Liquid Mixtures: Theory and Experiment. *ChemPhysChem.* 11:3722–3733.
37. Dai, J., J. Zhang, …, D. Grischkowsky. 2004. Terahertz Time-Domain Spectroscopy Characterization of the Far-Infrared Absorption and Index of Refraction of High-Resistivity, Float-Zone Silicon. *J. Opt. Soc. Am. B.* 21:1379–1386.
38. Ronne, C., P.-O. Astrand, and S. R. Keiding. 1999. THz Spectroscopy of Liquid H2O and D2O. *Phys. Rev. Lett.* 82:2888–2891.
39. Francis, G., Z. Kerem, …, K. Becker. 2002. The Biological Action of Saponins in Animal Systems : A Review. *Br. J. Nutr.* 88:587–605.
40. Figard, L., and A. M. Sokac. 2014. A Membrane Reservoir at the Cell Surface: Unfolding the Plasma Membrane to Fuel Cell Shape Change. *BioArchitecture.* 4:39–46.
41. Groulx, N., F. Boudreault, …, R. Grygorczyk. 2006. Membrane Reserves and Hypotonic Cell Swelling. *J. Membr. Biol.* 214:43–56.
42. Masters, T. A., B. Pontes, …, N. C. Gauthier. 2013. Plasma Membrane Tension Orchestrates Membrane Trafficking, Cytoskeletal Remodeling, and Biochemical Signaling during Phagocytosis. *Proc. Natl. Acad. Sci. USA.* 110:11875–11880.
43. Venkova, L., A. S. Vishen, …, M. Piel. 2022. A Mechano-Osmotic Feedback Couples Cell Volume to the Rate of Cell Deformation. *Elife.* 11:e72381.

Biophysical Journal, Volume *123*

Supplemental information

Impact of trypsin on cell cytoplasm during detachment of cells studied by terahertz sensing

Blandine Lordon, Tiffany Campion, Laure Gibot, and Guilhem Gallot

Supplementary Information

Impact of trypsin on cell cytoplasm during detachment of cells studied by terahertz sensing,
by B. Lordon, T. Campion, L. Gibot and G. Gallot

Relationship between the terahertz permittivity and the size of the in biomolecules

Figure SI1 shows the influence of the molar mass of the molecules in solution on the terahertz ATR signal. The data and the theoretical model are taken from the Supplementary Information of [1] and from [2] for the molecules listed in Table SI1. The solid line is obtained from a 3-volume model presented in [1]. It consists in assigning a terahertz permittivity to 3 different volumes: the molecule itself, the solvation shell around the molecule and the bulk water. The data show a strong dependence of the terahertz signal on the molar mass of the molecules. The contribution is negative for the smallest molecules below about 110 Da, whereas it is positive above that, for amino acids, peptides and proteins.

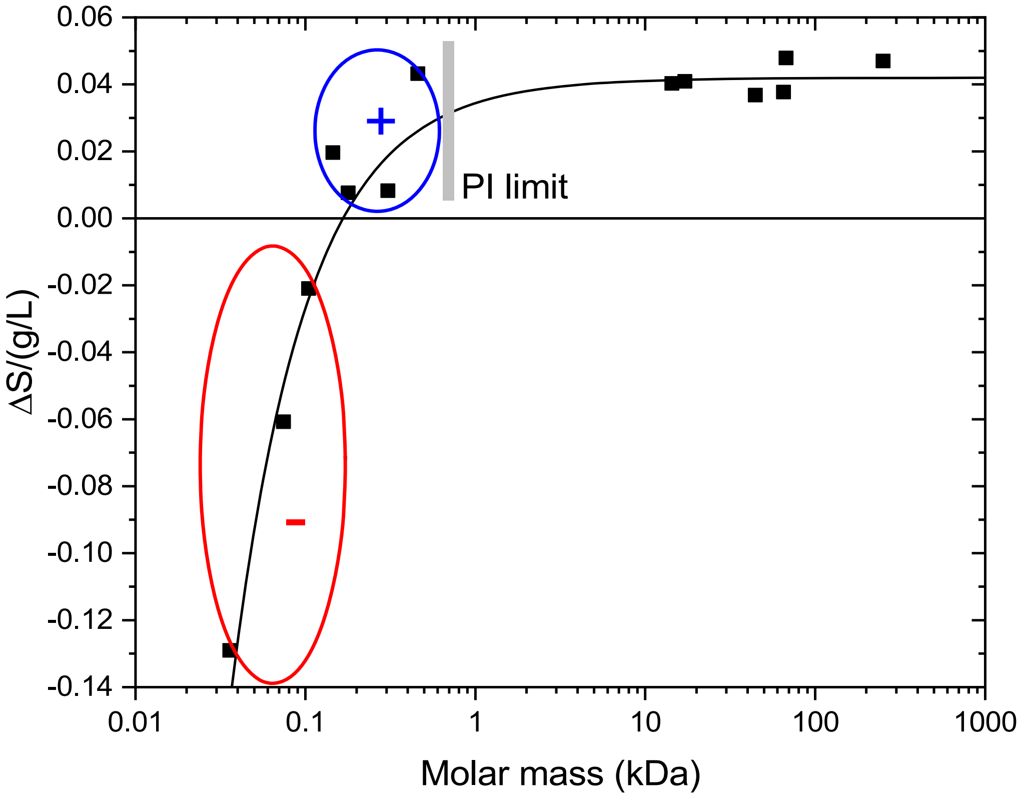

Figure SI1: Experimental and theoretical terahertz ATR reflectivity variation from biological relevant solutions found in Table SI1. ΔS/(g/L) is the molar variation of terahertz signal. The solid line is obtained from the model described in [1].

Molecule	Molar mass (kDa)
K^+/Cl^-	0.037
glycine	0.075
serine	0.105
lysine	0.146
tricine	0.179
gluthatione	0.307
LSKL-NH2 peptide	0.458
lysozyme	14.3
myoglobin	17.1
ovalbumin	44.3
hemoglobin	64.5
albumin	66
catalase	≈250

Table SI1: List of the molecules recorded in figure SI1, with their molar mass.

Contributions to the terahertz signal

The cytoplasm is experimentally found to have a positive contribution to the terahertz signal, while the PBS consists of small inorganic ions that have a negative contribution. Therefore, there are two possible explanations for a decrease in the terahertz signal, possibly at the same time. It could be due to an uptake of molecules from the PBS (see Table SI2 for the composition of the PBS) and/or an output of molecules from the cytoplasm with a molar mass between about 100 and 600 Da, as shown in Fig. SI2.

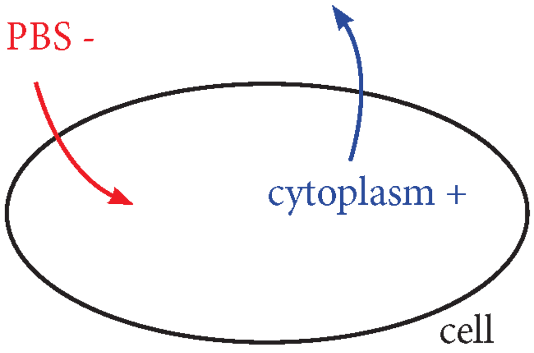

Figure SI2: Fluxes of molecules contributing to a decrease of the terahertz signal: influx from the PBS or output from the cytoplasm.

Molecules	Concentration mg/L
KH_2PO_4	144
NaCl	9000
Na_2HPO_4-$7H_2O$	795

Table SI2: Composition of PBS, pH 7.4, ThermoFisher Scientific, 10010023.

Videomicroscopy data

The monitoring by videomicroscopy of cell morphology and plasma membrane permeability (propidium iodide, red fluorescence) of MDCK1 cells incubated with trypsin (8µM) are presented in the two following movies: control and trypsin/EDTA.

Movie1-control

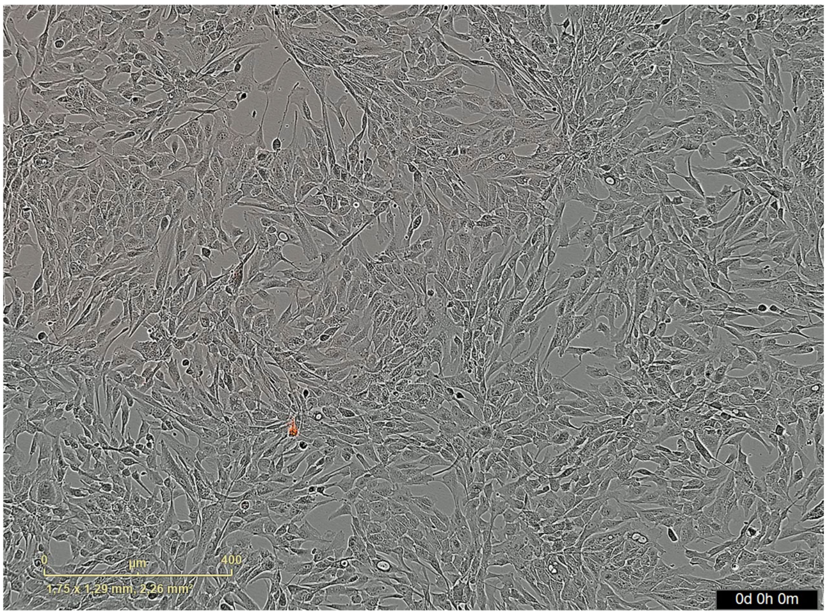

Movie1-trypsin/EDTA

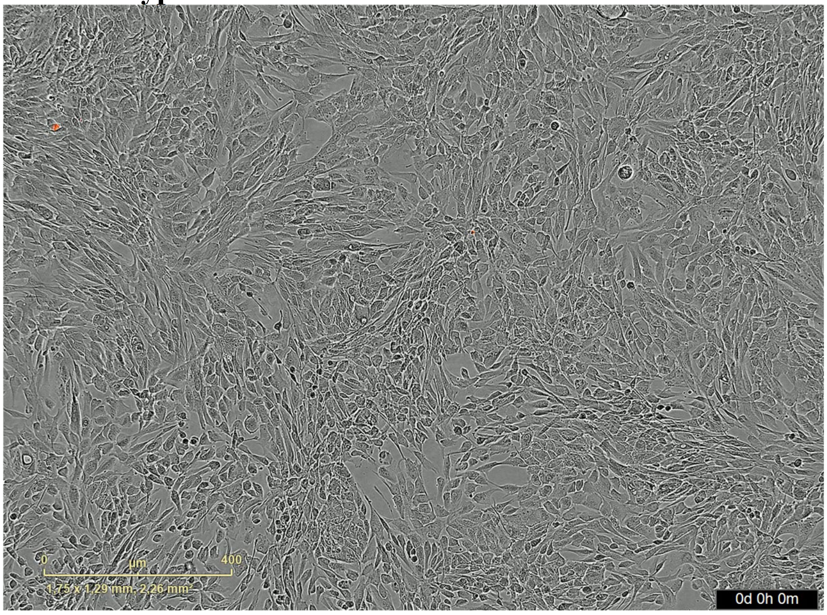

References:

[1] M. Grognot and G. Gallot, "Relative Contributions of Core Protein and Solvation Shell in the Terahertz Dielectric Properties of Protein Solutions", J. Phys. Chem. B 121, 9508 (2017).
[2] M. Grognot, "Imagerie térahertz par réflexion interne totale pour la biologie. Application à l'étude de la perméabilisation cellulaire", PhD thesis, Ecole polytechnique (2016).
